# Predicting Physical Activity and Healthy Nutrition Behaviors Using Social Cognitive Theory: Cross-Sectional Survey among Undergraduate Students in Chongqing, China

**DOI:** 10.3390/ijerph14111346

**Published:** 2017-11-05

**Authors:** Xianglong Xu, Yang Pu, Manoj Sharma, Yunshuang Rao, Yilin Cai, Yong Zhao

**Affiliations:** 1 School of Public Health and Management, Chongqing Medical University, Chongqing 400016, China; xianglong1989@126.com (X.X.); puyang79@126.com (Y.P.); rys0606@163.com (Y.R.); caiyilin95@126.com (Y.C.); 2 Research Center for Medicine and Social Development, Chongqing Medical University, Chongqing 400016, China; 3 The Innovation Center for Social Risk Governance in Health, Chongqing Medical University, Chongqing 400016, China; 4 Department of Behavioral and Environmental Health, Jackson State University, Jackson, MS 39213, USA; manoj.sharma@jsums.edu; 5 School of Nursing, Chongqing Medical University, Chongqing 400016, China

**Keywords:** physical activity, nutrition behaviors, social cognitive theory, expectations, self-efficacy, self-control, undergraduates, China

## Abstract

(1) Background: Generally suggested public health measures to reduce obesity were to limit television (TV) viewing, enhance daily physical activities, enable the consumption of fruit and vegetables, and reduce sugar-sweetened beverage intake. This study analyzed the extent to which selected social cognitive theory constructs can predict these behaviors among Chinese undergraduate students. (2) Methods: This cross-sectional study included 1976 undergraduate students from six universities in Chongqing, China. A self-administered five-point Likert common physical activity and nutrition behavior scale based on social cognitive theory was utilized. (3) Results: This study included 687 (34.77%) males and 1289 (65.23%) females. A total of 60.14% of the students engaged in exercise for less than 30 min per day. Approximately 16.5% of the participants spent at least 4 h watching TV and sitting in front of a computer daily. Approximately 79% of the participants consumed less than five cups of fruit and vegetables daily. Undergraduate students who had high self-efficacy scores had more leisure time physical activities. Those who have high expectation scores had considerable time watching TV and sitting in front of a computer. Undergraduate students who had high expectation and self-efficacy scores had substantially low consumption of sugar-sweetened beverages. Those who had high self-efficacy scores consumed considerable amounts of fruit and vegetables. Furthermore, the type of university, BMI group, gender, age, lack of siblings, and grade level were associated with the aforementioned four behaviors. (4) Conclusion: Physical inactivity and unhealthy nutrition behaviors are common among undergraduate students. This study used social cognitive theory to provide several implications for limiting the TV viewing, enhancing daily physical activities, consuming fruit and vegetables, and reducing sugar-sweetened beverage intake among undergraduate students.

## 1. Introduction

Physical inactivity and unhealthy nutrition behaviors have resulted in innumerable chronic diseases around the world. Generally suggested public health measures to reduce obesity were to limit television viewing, encourage moderately intense daily physical activities, increase fruit and vegetable intake, and increase water consumption [1]. The World Health Organization (WHO) asserted that physical inactivity causes numerous cases of cancer, diabetes mellitus, and cardiovascular diseases, thereby directly and indirectly killing 3.2 million people globally [2,3]. Sedentary behaviors, such as viewing television (TV) and using a computer, can potentially increase the risk of being overweight and obese and having colorectal cancer among adolescents [4,5]. Drinking insufficient water has been associated with population mortality [6]. Obesity, Type 2 diabetes, and cardio-metabolic diseases are related to the high consumption of sugar-sweetened beverages [7,8]. Approximately 2.8% of deaths globally are related to low consumption of fruit and vegetables [9].

Physical inactivity and unhealthy nutrition behaviors are common among university students. Many young people, particularly students, lack adequate exercise and fail to develop healthy nutrition behaviors. A quarter of adults globally are physically inactive and over 80% of adolescents do not engage in sufficient physical activities (i.e., at least 60 min of moderate- to vigorous-intensity daily physical activities) [10]. Over the past two decades, screen-based communication and entertainment have gained popularity among young people [11]. Sugar-sweetened beverages have been substantially consumed in many countries [12,13]. In 2006, the weekly physical activities of adults in China decreased by an average of 32% compared with that in 1991 [14]. The “2010 National Physical Fitness and Health Surveillance” asserted that 77.3% of primary and middle school students failed to meet the recommended levels of physical activities [15]. A survey corroborated that 20.8% of Chinese college students spent over 2 h per day in front of a screen [16]. In 2011, the proportions of daily vegetable and fruit intake were 321.6 g/d and 90.1 g/d, respectively, among the young and middle-aged residents (18–44 years old) of 9 provinces in China [17]. Vegetable consumption significantly declined and fruit consumption continuously increased in 2011 more than those in 1991 [17].

Social cognitive theory has been applied to the Chinese people for HIV prevention [18], smoking cessation [19], and obesity prevention [20,21]. To our knowledge, the current study is the first that focuses on predicting physical activities and healthy nutrition behaviors using social cognitive theory among undergraduate students in China. Social cognitive theory provides an advantageous framework for designing primary prevention interventions to reduce cases of obesity among the Chinese. Self-efficacy describes a person’s confidence in exhibiting a particular behavior at a given moment [22]. Self-control describes a person’s capability to regulate behavior and includes strategies that encourage proximal and distal goal-setting and self-rewards [22,23]. Expectations are the anticipation of the outcome of a particular behavior and the value that one places on these outcomes [23]. Hence, understanding social influences that affect health-risk behaviors may lead to the development of educational and environmental strategies that could influence the risky behaviors of undergraduate students. Health-risk behaviors, such as physical inactivity, poor eating habits, and smoking, are generally established during adolescence and young adulthood [24]. Social cognitive theory includes individual and environmental factors [25]. The current study analyzed the extent to which selected social cognitive theory constructs can predict these behaviors among Chinese undergraduate students and provide a scientific basis for obesity prevention and control. In particular, this study determined the role of expectations, self-efficacy, and self-control on leisure time physical activity behavior, TV and computer viewing behavior, fruit and vegetable intake behavior, and sugar-sweetened beverage consumption behavior among Chinese undergraduate students.

## 2. Materials and Methods

### 2.1. Research Method

A cross-sectional survey of undergraduate students was conducted in Chongqing in July 2015. Universities were classified and divided (depending on their respective specializations) into medical, normal, science and engineering, and comprehensive universities. Six universities were selected from all universities in Chongqing. The selected representative universities include a medical university (Chongqing Medical University and Chongqing Medical and Pharmaceutical College), normal university (Chongqing Normal University), engineering university (Chongqing University of Science and Technology), and comprehensive university (Chongqing University, Southwest University). All subjects gave their informed consent for inclusion prior to their participation in this study. The current study protocol was approved by the Ethics Committee of Chongqing Medical University (2013036).

### 2.2. Setting and Samples

Convenience sampling was used to select the students in the classrooms in the teaching building of each of the six universities. The investigating team members explained the research objectives and distributed the questionnaire to the students, who gave their written consent to participate. The students who consented were asked to complete the questionnaire in the classroom. The respondents were given approximately 15 min to complete the questionnaire. Prior to the collection of the questionnaire, the teams in charge of data collection were trained to obtain familiarity with the goals and methods of this study. They were also given specific instructions on how to fill out the questionnaire. During the data collection, the participants were randomly selected from each classroom in the teaching buildings. The participants who were willing to participate completed the questionnaire titled “Assessment of Healthy Behaviors: Surveys”. A total of 2100 completed questionnaires were collected from the original total of 2200 questionnaires. The preliminary response rate was 95.5%. Among the 2100 respondents, 1976 answered all the questions. The final sample included in the analysis comprised 1976 respondents. The study population consisted of 687 (34.77%) males and 1289 (65.23%) females.

### 2.3. Questionnaire

The questionnaire was developed by research analysts from Jackson State University and Chongqing Medical University. The questionnaire was translated from English into Chinese through translation and retranslation and was adopted to a Chinese context. The internal consistency of the section of physical activity and healthy nutrition behaviors based on social cognitive theory was acceptable (Cronbach’s alpha = 0.96).

#### 2.3.1. Demographic Variables

Demographic information included the type of university, gender, age, nationality, height, weight, current residence, grade point average credit, grade level, and lack of siblings. Self-reported height and weight data were collected to calculate the body mass index (BMI).The participants were classified into the following BMI groups [26]: low (BMI < 18.5), normal (18.5 ≤ BMI < 24), overweight (24 ≤ BMI < 28), and obese (BMI ≥ 28) groups.

For multivariable analysis, the influencing factors are leisure time physical activities, time of watching TV and sitting in front of a computer, amount fruit and vegetables consumed, and amount of sugar-sweetened beverages consumed. The type of university was categorized as medical university (Chongqing Medical University and Chongqing Medical and Pharmaceutical College) and non-medical university (Chongqing Normal University, Chongqing University, Chongqing University of Science and Technology, and Southwest University).

#### 2.3.2. Physical Activity and Healthy Nutrition Behavior Status

This study measured the status of physical activities and healthy nutrition behaviors of college students in Chongqing. All participants were asked to answer the following questions.

*a.*  Physical activity and healthy nutrition behavior status

(1) The survey included a question on self-reported exercise time.“From yesterday to today, how long did you exercise? (moderate-intense exercise/strenuous exercise)” was a closed question in which the participants reported the hours of exercise.
(2) The survey included two questions on the self-reported hours of watching TV and sitting in front of a computer. “From yesterday to today, how many minutes did you watch TV?” and “From yesterday to today, for how many minutes did you sit in front of a computer screen?” were closed questions in which the participants reported the number of hours of their screen time.
(3) The survey included two questions on self-reported water and sugar-sweetened beverage consumption. “From yesterday to today, how many bottles of water and sugar-free drinks did you consume?” and “From yesterday to today, how many bottles of sugary drinks did you consume?” were closed questions in which the interviewees reported the bottles of water and sugar drink intake, respectively.
(4) The survey included two questions on self-reported fruit and vegetable intake. “From yesterday to today, what was the weight of the fruits you ate?” and “From yesterday to today, what was the weight of the vegetables you ate?” are closed questions in which the interviewees reported the weight of fruit and vegetables.

*b.*  *Constructs of physical activity and healthy nutrition behaviors based on social cognitive theory* Constructs of social cognitive theory questions on physical activity and healthy nutrition behaviors based was derived from previous studies [27,28]

(1) Expectations, self-efficacy, and self-control for exercise for 30 min daily.
(2) Expectations, self-efficacy, and self-control for watching TV and sitting in front of a computer for <4 h.
(3) Expectations, self-efficacy, and self-control for drinking water instead of sugar-sweetened beverages.
(4) Expectations, self-efficacy, and self-control for eating at least five cups of fruit and vegetables (see Supplement File 1).

### 2.4. Data Analysis

Frequencies and percentages were calculated to summarize the distributions of the categorical variables. A t-test was employed to compare the differences in the continuous variables between males and females. Generalized linear models were developed using social cognitive theory variables (i.e., expectations, self-efficacy, and self-control), type of university, BMI groups, gender, age, lack of siblings, residence, grade level, and nationality as independent variables; and leisure time physical activity, time of watching TV and sitting in front of a computer, amount of fruit and vegetables consumed, and amount of sugar-sweetened beverages consumed as dependent variables. Statistical tests included a two-sided test and statistical significance was considered at *p* < 0.05. Data were analyzed using Statistical Analysis System software (version 9.1.3; SAS Institute, Cary, NC, USA).

## 3. Results

### 3.1. Characteristics of the Sample

This survey involved 2100 undergraduate students. A total of 1976 persons, which include 687 (34.77%) males and 1289 (65.23%) females, answered all the questions. All the respondents were between 16 to 26 years old. A total of 1861 (94.18%) were Han nationals and 115 (5.82%) were minorities. A total of 1925 (97.42%) lived on campus, 40 (2.02%) lived in apartments, and 11 (0.56%) lived at home. A total of 776 (39.27%) were Grade 1 students, 779 (39.42%) were Grade 2 students, 377 (19.08%) were Grade 3 students, and 44 (2.23%) were Grade 4 students. A total of 382 (19.33%) were from medical universities and 1594 (80.67%) were from non-medical universities. A total of 491 (24.85%) were low-weight, 77 (3.90%) were overweight, and 35 (1.77%) were obese. Approximately 43.62% (862) had no siblings and 56.38% (1114) had siblings. A total of 1189 (60.14%) students engaged in exercise for less than 30 min. A total of 327 (16.54%) participants spent at least 4 h watching TV and using a computer. A total of 992 (50.20%) consumed at least one bottle of sugar-sweetened beverage daily. A total of 1567 (79.26%) participants consumed less than five cups of fruit and vegetables (see Table 1).

### 3.2. Descriptive Statistics of Social Cognitive Theory Constructs for the Four Behaviors

Table 2 presents the means and standard deviations of the scores of the social cognitive theory constructs, namely, expectations to engage in the four behaviors, self-efficacy for performing the four behaviors, and self-control for performing the four behaviors. All standardized Cronbach’s alpha values were >0.8, except for self-efficacy for watching TV and using a computer for <4 h. The total standardized Cronbach alpha was 0.96 (see Table 2).

### 3.3. Social Cognitive Theory Assessment of Physical Activity and Healthy Nutrition Behaviors by Gender

Compared with males, females had a significantly higher mean score of expectations for drinking water instead of sugar-sweetened beverage (*p* = 0.0001), expectations for eating at least five cups of fruit and vegetables (*p* < 0.0001), and self-efficacy for drinking water instead of sugar-sweetened beverages (*p* < 0.0001). Compared with females, males had a significantly higher mean score of self-efficacy for exercise for 30 min daily (*p* < 0.0001) and self-control for exercise for 30 min daily (*p* < 0.0001). However, no significant differences between males and females were observed in the mean score of expectations for exercise for 30 min daily, expectations for watching TV and using a computer for <4 h, self-efficacy for watching TV and using a computer for <4 h, self-control for watching TV and using a computer for <4 h, self-control for drinking water instead of sugar-sweetened beverages, self-efficacy for eating at least five cups of fruit and vegetables, and self-control for eating at least five cups of fruit and vegetables (see Table 3).

### 3.4. Correlation Analyses of Physical Activity, Healthy Nutrition Behaviors, and Social Cognitive Theory Constructs

A statistically significant positive correlation was observed between self-efficacy and exercise status (Pearson’s correlation coefficient = 0.29, *p* < 0.001). A statistically significant positive correlation was observed between self-control for exercise and exercise status (Pearson’s correlation coefficient = 0.19, *p* < 0.001). A statistically significant negative correlation was observed between self-efficacy and screen time status (Pearson’s correlation coefficient = −0.14, *p* < 0.001). A statistically significant negative correlation was observed between self-control and screen time status (Pearson’s correlation coefficient = −0.13, *p* < 0.001). A statistically significant negative correlation was observed between expectations and beverage (Pearson’s correlation coefficient = −0.18, *p* < 0.001). A statistically significant negative correlation was observed between self-efficacy and beverage (Pearson’s correlation coefficient = −0.28, *p* < 0.001). A statistically significant negative correlation was observed between self-control and beverages (Pearson’s correlation coefficient = −0.18, *p* < 0.001). A statistically significant positive correlation was observed between expectations and fruit and vegetable status (Pearson’s correlation coefficient = 0.05, *p* = 0.020). A statistically significant positive correlation was observed between self-efficacy and fruit and vegetable status (Pearson’s correlation coefficient = 0.26, *p* < 0.001). A statistically significant positive correlation was observed between self-control and fruit and vegetable status (Pearson’s correlation coefficient = 0.20, *p* = 0.000) (see Table 4).

### 3.5. Multiple Linear Regression Analysis for the Factors Affecting the Social Cognitive Theory Constructs of Physical Activity and Healthy Nutrition Behaviors

In multivariable analyses, expectations, self-efficacy, and self-control were associated with leisure time physical activities, time of watching TV and sitting in front of a computer, amount of fruit and vegetables consumed, and amount of sugar-sweetened beverages consumed among Chinese undergraduate students (see Table 5). Students who had high self-efficacy scores had considerable leisure time physical activities. Students who had high expectation scores had substantial time watching TV and sitting in front of a computer. Students who had high expectations and high self-efficacy scores consumed limited sugar-sweetened beverages. Students who had high self-efficacy scores consumed considerable amounts of fruit and vegetables. Furthermore, multiple linear regression analysis obtained the following findings. Medical university students consumed more fruit and vegetables than non-medical university students. Male students had more leisure time physical activities, consumed more sugar-sweetened beverages, and consumed less fruit and vegetables than female students. Older students consumed substantial amount of fruit and vegetables. Students with low weight had less time of watching TV and sitting at a computer than students with normal weight. Students without siblings consumed more sugar-sweetened beverages than those with siblings. Grade 2 students had limited leisure time physical activities but had substantial time watching TV and sitting in front of a computer. Grade 3 students had limited leisure time physical activities, had considerable time watching TV and sitting in front of a computer, and consumed limited sugar-sweetened beverages. Grade 4 students had substantial time watching TV and sitting in front of a computer.

## 4. Discussion

Physical inactivity and unhealthy nutrition behaviors are common among undergraduate students. This study also determined that the constructs of selected social cognitive theory (i.e., expectations, self-efficacy, and self-control) can predict the four behaviors of limiting television viewing, enhancing physical activity, increasing fruit and vegetable intake, and limiting sugar-sweetened beverage consumption in undergraduate students. Moreover, this current study determined that the type of university, BMI groups, gender, age, lack of siblings, and grade level were associated with these four behaviors. Further health education and promotion measures to limit television viewing, encourage physical activities, increase fruit and vegetable intake, and limit sugar-sweetened beverage consumption among undergraduate students can enhance the expectations, self-efficacy, and self-control; and consider demographic characteristics, such as type of university, BMI groups, gender, age, lack of siblings, and grade level.

This study found that more than sixty percent of undergraduate students reported that they engaged in exercise for less than 30 min. WHO and The Chinese Dietary Guidelines (2016) recommends that adults should perform moderate-intensity aerobic physical activities for at least 150 min every week or moderate-intensity physical activities for 30 min daily [29,30]. Moreover, students who had high self-efficacy had substantial leisure time physical activity, which is similar to a previous study [31,32]. Physical activity intervention programs could consider enhancing self-efficacy for exercise, particularly for female undergraduate students and those from higher grade levels. To enhance self-efficacy, health education workers can conduct action planning, reinforce effort or progress toward physical activities, and provide instruction [33,34]. A previous study determined through training courses that self-efficacy substantially increased and that the daily consumption of cigarettes substantially decreased among middle-age Chinese [19]. Therefore, self-efficacy can be improved by providing students with training courses on physical activities.

Approximately sixteen percent of undergraduate students reported that they spent at least 4 h watching TV or using a computer, which is lower than the hours spent by American students in 2013 [35]. Most undergraduates surveyed in this study probably lived on campus and most universities did not provide TV in student dormitories. Therefore, they had limited opportunities to watch TV. The current study found that those who had high expectation scores had more time watching TV or sitting in front of a computer. A previous study showed that self-efficacy could predict the hours of TV watching among Chinese children [20]. This finding is different from our findings among undergraduate students. Health education workers could enhance expectations for watching TV and sitting in front of a computer for <4 h to reduce screen time among undergraduate students, particularly those from higher grade levels.

This study found that about half of students consumed at least one bottle of sugar-sweetened beverage daily. Moreover, the current study determined that those who had high expectation scores for drinking water instead of sugar-sweetened beverages and high self-efficacy scores for drinking water instead of sugar-sweetened beverages consumed limited amount of sugar-sweetened beverages. A previous study showed that those with high self-efficacy scores consumed limited soft drinks; the finding was similar to that of the present study [36]. Females consumed less amounts of sugar-sweetened beverages than males; this finding was similar to the study that indicated that males had higher probabilities of drinking sugar-sweetened beverages than females [37]. In this regard, self-efficacy can be built by prompting the review of behavioral goals, practice, and self-monitoring of behavior [33,34]. Measures are needed to enhance the expectations and self-efficacy for drinking water instead of sugar-sweetened beverages among Chinese undergraduate students, particularly students who are males, from lower grade levels, and without siblings.

This study determined that about eighty percent of students consumed below five cups of fruit and vegetables. Previous studies have shown that of the low consumption of fruit and vegetable was positively associated with the risk of cardiovascular diseases [38,39]. The current study determined that those who had high self-efficacy scores consumed substantial amounts of fruit and vegetables. This finding is similar to those of previous studies [40]. Moreover, previous studies confirmed that females consumed more fruit and vegetables than males [41,42]. This result is also reflected in the present study. Self-efficacy can be enhanced via practicing the behavior, monitoring behavior, and providing instruction [33,34]. To encourage undergraduate students to consume additional fruit and vegetables daily, self-efficacy for eating at least five cups of fruit and vegetables should be strengthened in future health education projects, particularly for those who are young, males, and from non-medical universities.

This study has several limitations that should be addressed. First, the sample may not represent all undergraduate students and the sample representation was insufficient for the research method. Accordingly, probability sampling was not employed. Second, the majority of the participants were females. Further studies should confirm and improve the applicability of the conclusion among male students. Third, this study focused only on the previous day, thereby possibly reflecting an unusual day and introducing bias. However, if a considerably long duration is considered, then such period can introduce recall bias and make the instrument cumbersome. Thus, a substantially short recall period was considered. Furthermore, this research is not a clinical instrument but a public health instrument that focuses on obtaining a broad image. Fourth, the survey was conducted through self-reporting and the information regarding health nutrition behaviors status may lead to response bias to the current findings. Watching TV and sitting in front of a computer for a long time and consuming sugar-sweetened beverages are universally acknowledged as unhealthy behaviors. Thus, students may have reported limited time of watching TV and sitting in front of a computer and limited amount of consumption of sugar-sweetened beverage. Lastly, the temporality of association cannot be determined given that this survey design was cross-sectional. Despite the aforementioned limitations, this study has a few advantages. This research is the first to explore the role of expectations, self-efficacy, and self-control for leisure time physical activity behaviors, TV and computer viewing behaviors, fruit and vegetable intake behavior, and limiting sugar-sweetened beverage consumption behavior among Chinese undergraduate students. This exploratory study provides a viable framework for the further exploration of the link among social cognitive theory, physical inactivity, and unhealthy nutrition behaviors issues, particularly in China. This method may facilitate addressing the issues of increasing physical inactivity and unhealthy nutrition behaviors of college students and provide a theoretical basis for intervention.

## 5. Conclusions

Prolonged TV viewing time, physical inactivity, diet that is low on fruit and vegetables, and high consumption of sugar-sweetened beverage are common among undergraduate students. This study determined the role of expectations, self-efficacy, and self-control on leisure time physical activity behavior, TV and computer viewing behavior, fruit and vegetable intake behavior, and sugar-sweetened beverage consumption behavior among Chinese undergraduate students. Furthermore, the type of university, BMI groups, gender, age, lack of siblings, and grade levels were associated with these four behaviors. This study provides a few implications for the four behaviors using social cognitive theory among undergraduate students in China.

## Figures and Tables

**Table 1:** Demographic characteristics of undergraduate students in Chongqing, China, 2015.

Variable	Number	Percentage or (Mean ± SD)
Gender		
Male	687	34.77
Female	1289	65.23
Age [16–26 years old]	1976	20.14 ± 1.32
Nationality		
Han nationals	1861	94.18
Minority	115	5.82
Residence		
Lives on campus or dormitory	1925	97.42
Lives in apartment or at home	51	2.58
Grade		
Grade 1	776	39.27
Grade 2	779	39.42
Grade 3	377	19.08
Grade 4	44	2.23
Type of university		
Medical university	382	19.33
Non-medical university	1594	80.67
BMI group		
BMI < 18.5 (low weight)	491	24.85
18.5 ≤ BMI < 24 (normal weight )	1373	69.48
24 ≤ BMI < 28 (overweight)	77	3.9
BMI ≥ 28 (obese)	35	1.77
Without siblings		
Yes	862	43.62
No	1114	56.38
Total time of daily exercise		
30 min and above	788	39.86
Below 30 min	1189	60.14
Total time of watching TV and sitting in front of a computer daily		
4 h and above	327	16.54
Below 4 h	1650	83.46
Total cups of sugar-sweetened beverage daily		
At least one bottle	992	50.2
None	984	49.8
Total cups of fruit and vegetables daily		
5 cups (200 mL per cup)and above	410	20.74
Below 5 cups	1567	79.26

**Table 2:** Social cognitive theory constructs of physical activity and healthy nutrition behaviors in undergraduate students in Chongqing, China, 2015.

Physical Activity and Healthy Nutrition Behaviors	Social Cognitive Theory Constructs	Minimum	Maximum	Mean	Std. Deviation	Standardized Cronbach Alpha
Exercise for 30 min daily	Expectations	9.00	45.00	33.33	6.40	0.88
	Self-efficacy	3.00	15.00	6.44	3.18	0.90
	Self-control	4.00	20.00	9.89	3.99	0.89
Watching TV and using a computer for <4 h	Expectations	10.00	50.00	37.01	6.90	0.88
	Self-efficacy	3.00	15.00	8.73	2.93	0.69
	Self-control	4.00	20.00	10.89	4.09	0.88
Drinking water instead of sugar-sweetened beverages	Expectations	10.00	50.00	36.84	7.26	0.89
	Self-efficacy	3.00	15.00	9.56	3.20	0.81
	Self-control	4.00	20.00	11.52	4.09	0.84
Eating at least five cups of fruit and vegetables	Expectations	10.00	50.00	38.67	7.35	0.90
	Self-efficacy	3.00	15.00	7.88	3.25	0.92
	Self-control	4.00	20.00	10.36	4.30	0.92
Total						0.96

**Table 3:** Constructs of social cognitive theory in undergraduate students by gender in Chongqing, China, 2015.

Physical Activity and Healthy Nutrition Behaviors	Social Cognitive Theory Constructs	Male	Female	*p*-Value
Exercise for 30 min daily	Expectations	33.54 ± 7.02	33.21 ± 6.04	0.303
	Self-efficacy	7.36 ± 3.34	5.94 ± 2.97	<0.001
	Self-control	10.70 ± 4.07	9.46 ± 3.87	<0.001
Watching TV and using a computer for <4 h	Expectations	36.89 ± 7.38	37.08 ± 6.62	0.583
	Self-efficacy	8.90 ± 3.02	8.64 ± 2.87	0.064
	Self-control	10.87 ± 4.12	10.91 ± 4.06	0.841
Drinking water instead of sugar-sweetened beverages	Expectations	35.99 ± 7.48	37.29 ± 7.10	0.0001
	Self-efficacy	9.17 ± 3.10	9.77 ± 3.23	<0.001
	Self-control	11.31 ± 4.13	11.63 ± 4.07	0.099
Eating at least five cups of fruit and vegetables	Expectations	37.61 ± 7.65	39.24 ± 7.12	<0.001
	Self-efficacy	7.71 ± 3.22	7.96 ± 3.27	0.104
	Self-control	10.17 ± 4.29	10.47 ± 4.30	0.144

**Table 4:** Correlation analysis of physical activity and healthy nutrition behaviors and social cognitive theory constructs.

Social Cognitive Theory Constructs	Leisure Time Physical Activity		Time of Watching TV and Sitting in Front of a Computer		Amount of Sugar-Sweetened Beverages Consumed		Amount of Fruit and Vegetables Consumed	
	Pearson Correlation Coefficient	*p*	Pearson Correlation Coefficient	*p*	Pearson Correlation Coefficient	*p*	Pearson Correlation Coefficient	*p*
Expectations	−0.03	0.360	0.05	0.170	−0.18	<0.0011	0.05	0.020
Self-efficacy	0.29	<0.001	−0.14	<0.001	−0.28	<0.001	0.26	<0.001
Self-control	0.19	<0.001	−0.13	<0.001	−0.18	<0.001	0.20	<0.001

**Table 5:** Multiple linear regression analysis for the factors that affect the social cognitive theory constructs of physical activities and healthy nutrition behaviors in Chongqing, China.

Parameter	Leisure Time Physical Activities			Time of Watching TV and Sitting in Front of a Computer			Amount of Sugar-Sweetened Beverages Consumed			Amount of Fruit and Vegetable Consumed		
	Estimate	Standard Error	*p*-Value	Estimate	Standard Error	*p*-Value	Estimate	Standard Error	*p*-Value	Estimate	Standard Error	*p*-Value
**Demographic characteristics**												
Medical university vs. Non-medical university	0.99	3.02	0.745	12.56	24.44	0.607	0.08	0.11	0.436	0.42	0.12	0.001
Males vs. Females	11.50	2.49	<0.001	12.68	20.76	0.542	0.41	0.09	<0.001	−0.66	0.11	<0.001
Age	1.94	1.20	0.106	−12.49	9.16	0.173	0.01	0.04	0.862	0.14	0.05	0.005
Han nationals vs. Minority	−3.25	4.98	0.515	27.56	39.53	0.486	−0.15	0.18	0.396	−0.39	0.21	0.062
Low weight vs. Normal weight	0.62	2.85	0.828	−52.10	22.93	0.023	−0.01	0.10	0.888	−0.11	0.11	0.339
Overweight vs. Normal weight	−8.11	5.95	0.173	−37.07	51.61	0.473	0.13	0.22	0.545	−0.08	0.25	0.743
Obese vs. Normal weight	4.07	8.31	0.625	22.49	65.35	0.731	0.17	0.32	0.604	−0.18	0.37	0.626
Lives off campus vs. Lives on campus	−4.23	8.64	0.625	−79.79	66.99	0.234	0.20	0.31	0.504	−0.57	0.35	0.104
Grade 2 vs. Grade 1	−6.58	2.86	0.022	53.53	23.56	0.023	−0.12	0.10	0.262	−0.17	0.12	0.159
Grade 3 vs. Grade 1	−10.43	4.06	0.010	65.43	31.93	0.041	−0.36	0.15	0.018	−0.07	0.17	0.692
Grade 4 vs. Grade 1	−17.89	9.10	0.050	130.36	63.97	0.042	−0.51	0.35	0.144	0.24	0.40	0.547
Without siblings vs. With siblings	0.48	2.40	0.842	18.95	19.86	0.340	0.18	0.09	0.039	−0.16	0.10	0.108
**Social cognitive theory constructs**												
Expectations	−0.32	0.19	0.084	3.07	1.40	0.028	−0.02	0.01	0.003	−0.01	0.01	0.093
Self-efficacy	3.52	0.54	<0.001	−8.67	4.51	0.055	−0.05	0.02	0.013	0.20	0.03	<0.001
Self-control	−0.19	0.45	0.677	−6.15	3.29	0.063	−0.002	0.01	0.911	−0.02	0.02	0.327

## Supplementary Materials

The following are available online at www.mdpi.com/1660-4601/14/11/1346/s1. File 1: Assessment of Healthy Behaviors: Survey.
