# Supplementary material for: Predicting Physical Activity and Healthy Nutrition Behaviors Using Social Cognitive Theory: Cross-Sectional Survey among Undergraduate Students in Chongqing, China
Source: Int J Environ Res Public Health. 2017 Nov 5;14(11):1346. doi: 10.3390/ijerph14111346 (PMC5707985; doi:10.3390/ijerph14111346)
Supplement: Supplementary file 1 [file ijerph-14-01346-s001.pdf]

Assessment of Healthy Behaviors: Survey

Demographic characteristics

1. Gender: Male or Female
2. What’s your age?
3. What’s your nationality? Han nationality/Minority
4. What’s your height?
5. What’s your weight?
6. What’s your current residence? Dormitory/Apartment/Home
7. What’s your Grade Point Average (GPA) credit?
8. What’s your subject?
9. What’s your grade?
10. Are you without siblings? Yes/No

Physical activity and healthy nutrition behavior status

1.From yesterday to today, how long did you exercise? (moderate-intense exercise/strenuous exercise)
2. From yesterday to today, how many minutes did you watch TV?
3. From yesterday to today, for how many minutes did you sit in front of a computer screen?
4. From yesterday to today, how many bottles of water and sugar-free drinks did you consume?
5. From yesterday to today, how many bottles of sugar drinks did you consume?
6. From yesterday to today, what was the weight of fruits that you ate?
7. From yesterday to today, what was the weight of vegetables that you ate?

Constructs of physical activity and healthy nutrition behaviors based on social cognitive theory of college students

1)Expectations for exercise for 30 minutes daily:

If I exercise for 30 minutes daily, I will: have few illnesses?/be highly confident?/have additional friends?/be extremely charming?/be able to study well?

Response options were “Never”, “Hardly Ever”, “Sometimes”, “Almost Always”, “Always”.

Which of the following changes are important to you: having few illnesses?/being highly confident?/having additional friends?/being extremely charming?

Response options were “Not At All Important”, “Slightly Important”, “Moderately Important”, “Very Important”, “Extremely Important”.

Self-efficacy for exercise for 30 minutes daily:

How sure are you that you will: exercise for 30 minutes daily?/insist on exercising for 30 minutes daily, even if you are tired?/insist on exercising for 30 minutes daily, even if you do not have time?

Response options were “Not At All Sure”, “Slightly Sure”, “Moderately Sure”, “Very Sure”, “Completely Sure”.

Self-control for exercise for 30 minutes daily: How sure are you that you will: set a goal to exercise for 30 minutes daily?/remind yourself to exercise for 30 minutes daily?/constantly check progress to make sure you exercise for 30 minutes?/reward yourself for insisting on exercising for 30 minutes daily?

Response options were “Not At All Sure”, “Slightly Sure”, “Moderately Sure”, “Very Sure”,

“Completely Sure”.

2) Expectations for watching TV for <4 hours and sitting at a computer:
If I spend less than 4 hours sitting at a computer screen, I will: have additional friends?/have extra free time?/be extremely pleasant?/be highly relaxed?/be able to study well?
Response option were “Never”, “Hardly Ever”, “Sometimes”, “Almost Always”, “Always”.
Which of the following changes are important to you: having additional friends?/having extra free time?/being extremely pleasant?/being highly relaxed?/being able to study well?
Response options were “Not At All Important”, “Slightly Important”, “Moderately Important”, “Very Important”, “Extremely Important”.
Self-efficacy for watching TV for <4 hours and sitting at a computer:
How sure are you that you will: reduce the time spent sitting at a computer and watching TV to less than 4 hours?/reduce the time of watching TV even if your favorite program is coming?/reduce the time of surfing the Internet even if you are supposed to do homework?
Response options were “Not At All Sure”, “Slightly Sure”, “Moderately Sure”, “Very Sure”, “Completely Sure”.
Self-control for watching TV for <4 hours and using a computer:
How sure are you that you will: set a goal that the time spent in watching TV and using a computer is less than 4 hours?/constantly check progress in making sure the time spent in watching TV and using a computer is less than 4 hours?/reward yourself for reducing the time spent in watching TV and using a computer to less than 4 hours?/remind yourself that time spent in watching TV and using a computer should be less than 4 hours?
Response options were “Not At All Sure”, “Slightly Sure”, “Moderately Sure”, “Very Sure”,” Completely Sure”.

3) Expectations for drinking water instead of sugar-sweetened beverages:
If I consume water and sugar-free instead of sugar-sweetened drinks, I will: be highly relaxed?/feel good?/be considerably dynamic?/have ideal weight?/be able to study well?
Response options were “Never”, “Hardly Ever”, “Sometimes”, “Almost Always”, “Always”.
Which one of the following changes are important to you: being highly relaxed?/feeling good?/being considerably dynamic?/having ideal weight?/being able to study well?
Response options were “Not At All Important”, “Slightly Important”, “Moderately Important”, “Very Important”, “Extremely Important”.
Self-efficacy for drinking water instead of sugar-sweetened beverages:
How sure are you that you will: consume water and sugar-free drinks instead of sugar-sweetened drinks?/even if water and sugar-free beverages are unpleasant to drink, you will consume them instead of sugar drinks?/drink plenty of water even if you do not feel extremely thirsty?
Response options were “Not At All Sure”, “Slightly Sure”, “Moderately Sure”, “Very Sure”, “Completely Sure”.
Self-control for drinking water instead of sugar-sweetened beverages:
How sure are you that you will: set a goal for consuming water instead of sugar drinks?/reward yourself for consuming water instead of sugar drinks?/remind yourself to consume water instead of sugar drinks daily?/constantly check progress in making sure you consume water instead of sugar drinks?

Response options were “Not At All Sure”, “Slightly Sure”, “Moderately Sure”, “Very Sure”, “Completely Sure”.

4)Expectations for 5 or more cups of fruits and vegetables:
If I eat more than 5 cups of vegetables and fruits, I will: be considerably dynamic?/feel well?/have few illnesses?/have ideal weight?/have abundant food?
Response options were “Never”, “Hardly Ever”, “Sometimes”, “Almost Always”, “Always”
Which one of the following changes are important to you: being considerably dynamic?/feeling well?/having few illnesses?/having ideal weight?/having abundant food?
Response options were “Not At All Important”, “Slightly Important”, “Moderately Important”, “Very Important”, “Extremely Important”.
Self-efficacy for consuming five or more cups of fruits and vegetables:
How sure are you that you will: eat more than five cups of vegetables and fruits daily?/eat more than five cups of vegetables and fruits daily, even if you do not like eating fruits and vegetables?/eat more than five cups of vegetables and fruits daily, even if the other family members do not like eating fruits and vegetables?
Response options were “Not At All Sure”, “Slightly Sure”, “Moderately Sure”, “Very Sure”, “Completely Sure”.
Self-control for consuming five or more cups of fruits and vegetables:
How sure are you that you will: set a goal to eat more than five cups of vegetables and fruits daily?/reward yourself for eating more than five cups of vegetables and fruits daily?/remind yourself to eat more than five cups of vegetables and fruits daily?/constantly check progress to make sure you eat more than five cups of vegetables and fruits daily?
Response options were “Not At All Sure”, “Slightly Sure”, “Moderately Sure”, “Very Sure”, “Completely Sure”.

健康行为评估调查

学校名称：
人口学问题
1.您的性别？ □ 男 □ 女
2.您的年龄？ ________________岁
3.您的民族？ □汉族 □少数民族（ 请填写）_________
4.您的身高？ （cm）
5.您的体重？ （kg）
6.您目前的生活地点？ □住校园，宿舍 □住校外，公寓 □住校外，家里
7. 您的累计平均学分绩点？ ____________
8. 您的专业？ ________________
9. 您的年级？ ________________
10. 您是否为独生子女？ □是 □否

1.从昨天到现在，您进行了多少分钟的锻炼？（请填写）
注：在本次调查中，锻炼是指每天进行 30 分钟及以上的中等强度及剧烈运动。
中等强度运动，包括如快走，打网球，缓慢地骑自行车，轻松地游泳，或跳舞等的可能会使您流汗但不会觉得疲惫的运动。

2.从昨天到现在，您看了多少分钟的电视？（请填写）______分钟
3.从昨天到现在，您花了是多少分钟坐在电脑屏幕前？（请填写）______分钟
4.从昨天到现在，您喝了多少杯（约 200ml 为一杯）含糖饮料？注：不包括任何的果汁。______杯
5.从昨天到现在，您吃了多少份（每份约 200g）水果？（请填写）______ 份
6.从昨天到现在，您吃了多少份（每份约 200g）蔬菜（请填写）______ 份

社会认知理论：
如果我每天锻炼 30 分钟，我会： 绝不 几乎不 有时 几乎总是 总是
1.少生病？
2.更自信？
3.有更多朋友？
4.变得更有魅力？
5.学习更好？
以下变化对您有多重要？ 一点也不重要 稍微有点重要 一般重要 很重要 非常重要
6.不经常生病？
7.更自信？
8.有更多朋友？
9.看起来精神？
您有多确定您会： 一点也不肯定 稍微有点肯定 一般肯定 很肯定 完全肯定
10.每天锻炼 30 分钟？
11.即使您累了仍然坚持每天锻炼 30 分钟？
12.即使您没有时间，仍然坚持每天锻炼 30 分钟？
您有多确定您会： 一点也不肯定 稍微有点肯定 一般肯定 很肯定 完全肯定
13.设立每天锻炼 30 分钟的目标？
14.提醒自己每天锻炼 30 分钟？
15.不断地检查进度以确保您每天锻炼 30 分钟？
16.因为坚持锻炼而奖励自己喜欢的东西？

2)如果我每天看电视和面对电脑的时间少于 4 小时，我会：
绝不 几乎不 有时 几乎总是 总是
17.有更多朋友？
18.有更多自由时间？
19.更愉快？
20.更轻松？
21.学习更好？
以下变化对您有多重要？ 一点也不重要 稍微有点重要 一般重要 很重要 非常重要
22.有更多朋友？
23.有更多自由时间？

24.更愉快？
25.更轻松？
26.学习更好？
您有多确定您会：　一点也不肯定　稍微有点肯定　一般肯定　很肯定　完全肯定
27. 每天看电视和面对电脑屏幕的时间不超过 4 个小时？
28. 即使您喜欢的节目到了，仍会减少看电视的时间？
29. 如临近提交作业的时间，也会减少上网时间？
您有多确定您会：　一点也不肯定　稍微有点肯定　一般肯定　很肯定　完全肯定
30. 为自己设立每天看电视和面对电脑屏幕的时间不超过 4 小时的目标？
31. 因为减少看电视和面对屏幕的时间而奖励自己喜欢的东西？
32. 提醒自己每天看电视和面对电脑屏幕的时间不超过 4 个小时？
33. 不断地检查您的进展以确保您每天看电视和面对电脑屏幕的时间不超过 4 个小时？

3)如果我喝水和无糖饮料，而不喝含糖饮料，我会：
绝不　几乎不　有时　几乎总是　总是
34.更轻松？
35.感觉更好？
36.更有活力？
37.有更理想的体重？
38.学习更好？
以下变化对您有多重要？　一点也不重要　稍微有点重要　一般重要　很重要　非常重要
39.更轻松？
40.学习更好？
41.感觉更好？
42.更有活力？
43.有更理想的体重？
您有多确定您会：　一点也不肯定　稍微有点肯定　一般肯定　很肯定　完全肯定
44. 用水和无糖饮料替代含糖饮料？
45. 即使不好喝，也会用水和无糖饮料替代含糖饮料？
46. 即使您不觉得渴每天也多喝水？
您有多确定您会：　一点也不肯定　稍微有点肯定　一般肯定　很肯定　完全肯定
47. 为自己设立每天喝水来替代含糖饮料的目标？
48. 因为用水来代替含糖饮料而奖励自己喜欢的？
49. 每天提醒自己喝水而不喝含糖饮料？
50. 不断地检查您的进展以确保您每天喝水而不喝含糖饮料？

4)如果我每天吃 5 份或以上的水果和蔬菜，我会：
绝不　几乎不　有时　几乎总是　总是
51.更有活力？

52.感觉更好？
53.少生病？
54.有更理想的体重？
55.我的食物更丰富？
以下变化对您有多重要？　一点也不重要　　　稍微有点重要　一般重要　很重要　　非常重要
56.不经常生病？
57.感觉更好？
58.更有活力？
59.有更理想的体重？
60.喜欢您的食物？
您有多确定您会：　一点也不肯定　　稍微有点肯定　　一般肯定　　很肯定　　　完全肯定
61.每天吃 5 份或以上的水果和蔬菜？
62.即使不喜欢，每天也会坚持吃 5 份或以上的水果和蔬菜？
63.即使家里其他人不喜欢，自己每天也会吃 5 份或以上水果和蔬菜？
您有多确定您会：一点也不肯定　　稍微有点肯定　　一般肯定　　很肯定　　　完全肯定
64.为自己设立每天吃 5 杯或以上的水果和蔬菜的目标？
65.因为每天吃 5 份或以上的水果和蔬菜而奖励您自己喜欢的？
66.提醒自己每天吃 5 份或以上的水果和蔬菜？
67.不断地检查您的进展以确保您每天吃 5 份或以上的水果和蔬菜？
